# Cardiac Mast Cells: A Two-Head Regulator in Cardiac Homeostasis and Pathogenesis Following Injury

**DOI:** 10.3389/fimmu.2022.963444

**Published:** 2022-07-15

**Authors:** Jing Jin, Yuanyuan Jiang, Subrata Chakrabarti, Zhaoliang Su

**Affiliations:** ^1^ International Genome Center, Jiangsu University, Zhenjiang, China; ^2^ Institute of Immunology, Jiangsu University, Zhenjiang, China; ^3^ Children’s Hospital of Nanjing Medical University, Nanjing, China; ^4^ Department of Pathology and Laboratory Medicine, Western University, London, ON, Canada

**Keywords:** cardiac mast cells, cardiac development, cardiac aging, cardiac injury, inflammation

## Abstract

Cardiac mast cells (CMCs) are multifarious immune cells with complex roles both in cardiac physiological and pathological conditions, especially in cardiac fibrosis. Little is known about the physiological importance of CMCs in cardiac homeostasis and inflammatory process. Therefore, the present review will summarize the recent progress of CMCs on origin, development and replenishment in the heart, including their effects on cardiac development, function and ageing under physiological conditions as well as the roles of CMCs in inflammatory progression and resolution. The present review will shed a light on scientists to understand cardioimmunology and to develop immune treatments targeting on CMCs following cardiac injury.

## Introduction

Mast cells, innate non-circulating immune cells, exist in almost all tissues and play key roles in allergic disease and host defense, including the heart. Mast cells are highly heterogeneous, express a range of receptors on their surface and generate a variety of mediators to involve in extensive inflammation and immune regulation through degranulation. Therefore, mast cells are called “sentinels” in harmful conditions, with the ability to rapidly perceive invasion and initiate immune defense and different biochemical programs of homeostasis in time (1). Cardiac mast cells (CMCs) are present in the heart at a low density at homeostasis and is generally detected in the epicardium, endocardium, and myocardium of ventricle and atrium in mice, rats, and humans. Published data show that CMCs density is <1 cell/mm^2^ in mouse heart (2). Our unpublished data also demonstrate that CMCs account for <3% of CD45^+^ cells in mouse heart. Therefore, the present review will summarize the novels progress of CMCs on origin, development, replenishment, especially on cardiac development, function and ageing under physiological conditions as well as the roles of CMCs on inflammatory progression and resolution.

## Origin, development and survival of CMCs

Mast cells have been always thought to originate from hematopoietic stem cells (HSCs) in bone marrow (3). However, recent data indicate that mast cells probably derive from three embryonic hematopoietic waves: early and late erythron-myeloid progenitors (EMPs) from yolk sac, and definitive HSCs from the aorta, gonads, and mesonephros region (4). Mast cells derived from different hematopoietic waves have different tissue preferences, for example, from the early EMPs distribute in adipose tissue, from late EMPs widely distribute in most connective tissues, and from fetal HSCs are the main cells group in mucosa. It is also suggested that bone marrow derived mast cells mainly replenish the mucosal mast cells (MMCs) after birth (4–6). Mast cells from the embryonic stage are thought to have reached peripheral tissues and matured into resident mast cells before birth, which possess tissue and function heterogeneity. After birth, mast cells precursors from bone marrow need to be released into the bloodstream and recruited by various mediators before entering the peripheral tissues. It is known that plenty of biologic agents, including growth factors, integrins, chemokines and adenosine nucleotides contribute to this recruitment process (7–10). Different mast cells subsets express different receptors which may contribute to their movement into specific tissues. For example, the recruitment to intestine requires α4β7 integrin and chemokine receptor CXCR2 expressed on mast cells progenitors (MCps), accompanying with mucosal addressin cellular adhesion molecule-1 and vascular cell adhesion molecule-1 on intestinal endothelium (11, 12). Furthermore, α4β7 and vascular cell adhesion molecule-1 are also required for the recruitment of mast cells precursors to the lungs (13). However, it is unclear that CMCs are derived from early and late EMPs, or maturation from MCp (14, 15). If it is the later, which factor can mediate the specific homing or recruitment of MCps to heart?

In both mouse and human, obtaining the cell surface and intracellular characteristics of fully differentiated mature mast cells requires a gradual process, that can be regulated by different cytokines, in which stem cell factor (SCF) and IL-3 may play a major role. SCF, the ligand of c-kit, not only facilitates cells migration, but also contributes to their development (16, 17). IL-3 can benefit the multiple hematopoietic lineage differentiation into mast cells *in vitro* (18, 19). However, IL-3 is not necessary for the generation of mast cells at homeostasis, it does benefit to increase the number (20). Like IL-3, the other cytokines, such as IL-4, IL-9, IL-10 and IL-13 can also synergistically promote mast cell proliferation and differentiation (21).

**Figure 1:**
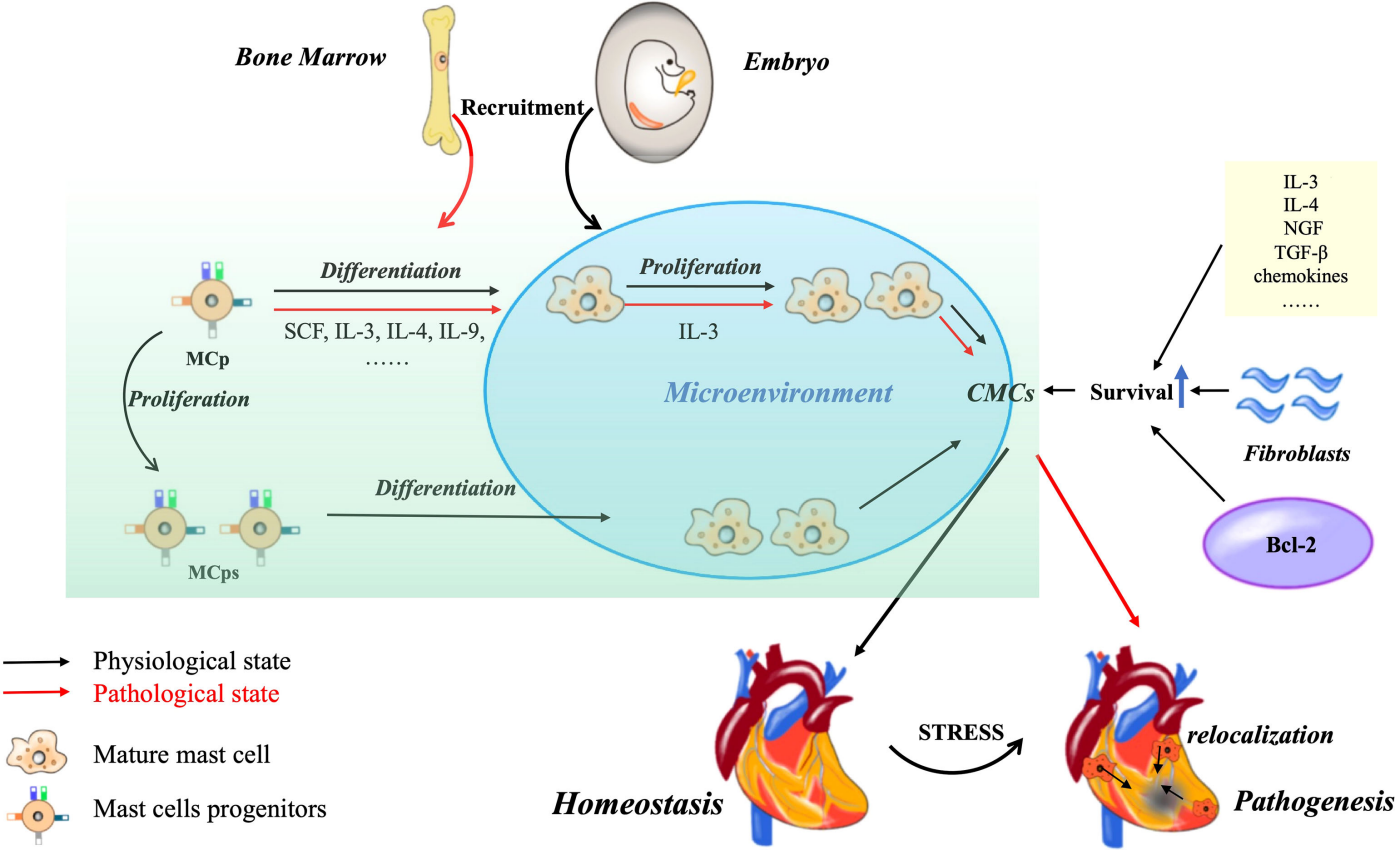
CMCs origin, development and survival. Most of CMCs in the physiological state of the heart come from embryonic stage, and only a small part come from bone marrow. The increase of CMCs can be differentiated from MCp or through self-proliferation. Under pathological conditions, MCps are recruited from bone marrow. The density can also be increased by the relocation of CMCs in non-injured sites. In addition, some cytokine chemokines, cardiac fibroblast derived growth factors and Bcl-2 family can promote the survival of CMCs.

Then, how are CMCs maintained and renewed? Tissue mast cells are known to be long-lived cells and even after degranulation they can re-granulate and continue to survive, which is dependent on the local SCF levels (22). Because SCF can inactivate FOXO3a, a fork-head transcription factor, and down-regulate and phosphorylate its target Bim (a Bcl-2 homology 3-only proapoptotic protein) which promote mast cells survival (23). Bcl-2 family, well-known proteins, are critical for cells survival and death. Christine Moller and his colleagues are the first to directly demonstrate that both *Bcl-x* and *Bcl-2* are essential for keeping mast cells survival during late development. Nevertheless, the upregulation of Bcl-XL and Bcl-2 by IgE is eliminated for bone marrow derived mast cells in IL-3^–/–^mice. IL-3 regulates pro-survival Bcl-2 family members (24, 25) and SCF suppresses pro-apoptotic Bim (23). The survival of mast cells depends on the ratio and interaction of anti-apoptotic and pro-apoptotic factors (26). Additionally, fibroblasts can also promote human mast cells survival (27). For example, it has been proved that mouse skin 3T3 fibroblasts can sustain the primary human lung mast cells for 13 days in the absence of exogenous growth factors (28). Although all the mast cells seemed not to have tight junctions and proliferation, these cells still maintained the general morphology, granule morphology and mediator content (29). Furthermore, the co-culture of gut-derived human mast cells with gut fibroblasts has the similar phenomena, and it is also indicated that human fibroblasts promote survival of human mast cells independent of SCF, IL-3, IL-4, and nerve growth factor (NGF). That’s because fibroblasts can release a soluble heat-sensitive molecule that downregulates apoptosis without promoting cell proliferation (30). In addition, an increase of mast cells is noted in the healing myocardium, and their progenitors are also found in the infarcted area, which is related to the activation and proliferation of fibroblasts following cardiac injury or not? The contribution of fibroblasts proliferation cannot be clarified, while the chemotaxis of circulating precursors to the heart may be the main mechanism leading to the accumulation of mast cells in ischemic heart (31). We at least know that fibroblasts contribute to mast cell survival. Other chemokines and cytokines involving in mast cells growth and survival need to be further confirmed in future (**Figure 1**) (32).

## Mast cells subsets

The classic classification of mast cells is based on their tissue distribution and granule content. According to the proteases they contained, mast cells are divided into mainly containing tryptase (MCTs) or chymase (MCCs) or both (MCTCs) (33). In human, almost 90% CMCs are MCTCs (34). MCTs is usually localized to mucosal surfaces and closely related to T cells, especially Th2-type. MCTCs, on the other hand, includes tryptase, chymase, carboxypeptidase and cathepsin G. It mainly exists in the gastrointestinal tract, skin, synovium and subcutaneous tissues. But the proportion and distribution of the two subsets may change in pathological states. For example, the number of MCTCs is increased in fibrotic diseases, but relatively unchanged in allergic or parasitic diseases. Therefore, MCTCs may be biased towards tissue remodeling and angiogenesis, and MCTs contribute to inflammation (35). Besides, mouse mast cells also can be divided into two lineages: inducible bone marrow–mucosal mast cells (MMCs) and constitutive embryonic-derived connective tissue mast cells (CTMCs) (36). Phenotypic differences between CTMCs and MMCs are acquired during the local tissue development, rather than determined by the genetic composition of their MCp or their different location in connective or mucosa tissue (37). In addition, like neutrophils (N1 and N2) and macrophages (M1 and M2), the complex biochemical environment of the tumor may promote mast cells differentiation into anti-tumor MC1 or pro-tumor MC2 (38). On the one hand, mast cells can generate excessive functionally active ROS which may induce cytotoxic effects that can promote tumor regression (39). On the other hand, large amounts of ROS exceed the capacity of cellular DNA repair systems, that may foster the occurrence of tumors. In addition, many other mast cell-derived mediators can also play distinct or even opposite roles in tumorigenesis (40).

## The microenvironment promotes formation of specific mast cells phenotype

Heterogeneity is a major feature of mast cells, reflecting the complex interaction between different microenvironmental signals transmitted by tissues and the differentiation programs that determine their phenotypes (41). However, how mast cells form a highly heterogeneous phenotype affected by microenvironment in peripheral tissues has rarely been mentioned. Generally, cells in a given population show heterogeneity, which means that once they show a certain minimum level of variation in one or more characteristics (42). The preliminary studies demonstrate that mast cells at different anatomical positions have significant morphological differences (43). Other studies also show that in addition to differing in morphologic, rat and mouse MMCs and CTMCs appear to differ in many other aspects of biochemistry, histochemical characteristics, function and roles in inflammation and immunity (44–46). Similarly, human mast cells also differ in various aspects of their phenotype, just like morphologic characteristics, histochemistry, contents of proteases and sensitivity to stimulation by secretogogues (47–51). Notably, it has been suggested that the phenotype of mast cells, such as mediator contents or responsive abilities to specific stimuli, can be regulated, at least in some cases reversibly, by microenvironmental signals such as cytokines and growth factor (52). In fact, many potential variations in microenvironment may affect phenotype. The anatomical location is the first factor that affects the phenotype. For example, when cultured mast cells *in vitro* were transferred into different locations *in vivo*, which can give the chance to develop into CTMCs or MMCs, depending on local signals (53, 54). Secondly, inflammatory or immune processes may also cause transient changes of mast cells phenotype. For instance, the number of CMCs increase in cardiomyopathy compared to normal myocardium, and a second increase occurred after long-term mechanical support, but the phenotype is conversion from MCTCs into MCTs with the decrease of cardiac fibrosis (55). Furthermore, similar switch also exists in other specific conditions, for example, T cell-dependent response may contribute to mast cells proliferation or maturation/differentiation, high concentrations of eosinophils may benefit the switch of mast cells from MMCs to MCTCs (42). Finally, mast cells may also participate in the regulation of their numbers and phenotypes, especially during inflammation or disease, by autocrine or paracrine or other potential mechanisms (56). For instance, IL-4 possessing growth factor activity for mast cells in mice can promote phenotypic conversion into CTMCs with IL-3 (57). The more detailed mechanisms of the microenvironment on phenotype still need to be explored. Single cell RNA sequencing data of mast cells will provide further insights into heterogeneity as well as clear views of differences between and within different tissues.

## CMCs distribution in heart and their functions

In mice, CMCs are mostly distributed in the epicardium (50%) or myocardium (45%), and a fraction is distributed in the endocardium (5%) (58). Similarly, the most CMCs of human are located in the interstitium and in the epicardium (59). Mast cells and their mediators are generally thought to participate in allergic diseases, however, increasing evidences suggest that mast cells may also play protective roles in several other pathological or physiological processes (59, 60). Single-Cell Sequencing shows that CMCs are existence in myocardium and epicardium, and activated and expanding in pressure overload-driven heart failure mouse model (61), furthermore, CMCs infiltration increase atrial fibrillation susceptibility following atrial burst stimulus (62). CMCs increase has been implicated in the chronic volume overload secondary to mitral regurgitation and aorto-caval fistula (63). Furthermore, mast cells in different site may possess the functional heterogeneity (60), for example, tryptase can activate protease-activated receptor 2 (PAR-2) located on cardiomyocytes, which may play a protective role during myocardial infarction (64). Moreover, PAR-2 on nerve fibers and myofibroblasts can also be activated by tryptase, which stimulates the release of substance P from sensory nerve fibers, which in turn activates MRGPRX2 receptors, a family of mas-related G-protein-coupled receptors, on human CMCs (1). The renin and chymase derived from the activated MRGPRX2 receptor, then respectively remove angiotensinogen and angiotensin I (Ang I) to form Ang II. The co-expression of renin and chymase by CMCs is very important for regulating the homeostasis of the cardiac renin-angiotensin system (59). Additionally, immunologic stimuli, bacterial and viral superantigens can activate primary human CMCs to release angiogenic (VEGF-A) and lymphangiogenic (VEGF-C) factors (1, 60, 65). Besides VEGF-A promoting angiogenesis, VEGF-C can also stablize blood pressure, promote lipid metabolism, and coronary artery development (60, 66–68).

## CMCs on cardiac development, senescence and function

Most reviews focus on the roles of mast cells in pathological conditions, the present review focuses on the physiological roles. A few studies have suggested that mast cells may participate in the morphogenesis of some mouse organs, such as the mammary glands (69) and corneal (70). The published data demonstrated that the density and number of CMCs are dynamically changed with age in rat (71). Our unpublished data also demonstrate that CMCs exist at embryonic stage in mouse heart. Therefore, we speculate that the CMCs may contribute to cardiac development. Similarly, CMCs density in children was low under the two years old, but the number of CMCs firstly increases and then decreases continuously with age (72). The rapid increase of CMCs density in the early postnatal period accompanies angiogenesis. Furthermore, the corneal mast cells promote corneal angiogenesis (73). All these data suggest that CMCs may play physiological roles on cardiac growth and development. In addition, immune-activated human CMCs can also produce VEGF-A and VEGF-C to induce the formation of new blood vessels and lymphatics, while the similar function in physiological state has not been confirmed (60, 68). CMCs exist not only around the cardiac vessels of neonatal mouse, but also around the nerve fibers. So CMCs might also have a positive effect on nerve development in heart (74, 75).

**Figure 2:**
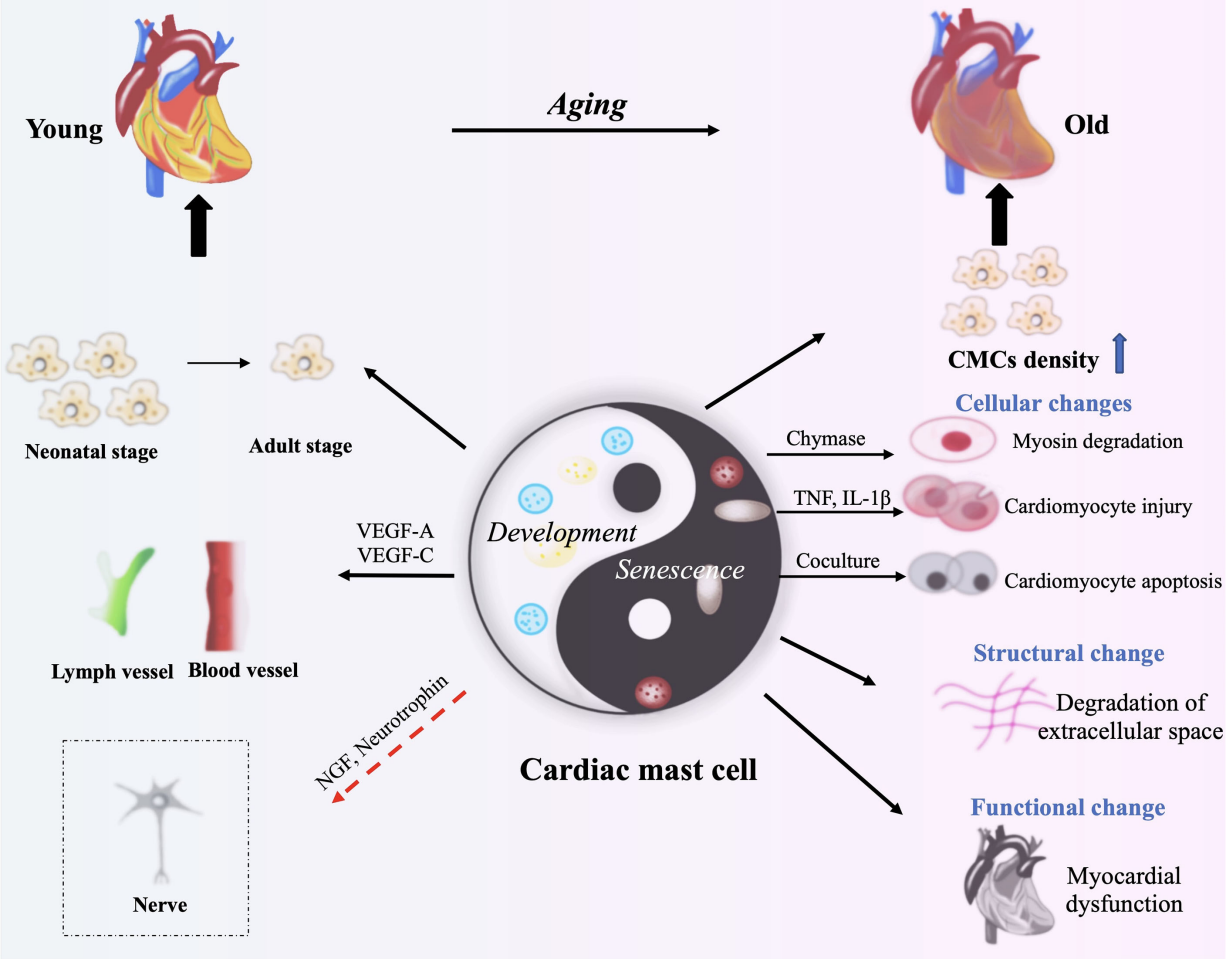
The roles of CMCs in cardiac development and aging. VEGF-A, VEGF-C, NGF and neurotrophin from CMCs contribute to cardiac development through benefiting the formation of blood vessels and lymphatic vessels, and the development of cardiac nerves (neonatal stage). CMCs produce Chymase, TNF and IL-1β to degrade myosin or to damage cardiomyocytes. CMCs’ activation following cardiac injury causes inflammatory response, and then lead to the structural damage and cardiac dysfunction (Old Stage). The red arrow means speculation.

Multiple evidences indicate that mast cells may be involved in the development of the heart, so whether it has an impact on the aging of the heart? Although there is no direct evidence that mast cells are involved in heart aging, we can speculate from the effect of mast cells on the aging of other tissues and organs. Firstly, the number of mast cells in the mesenteric lymphatic vessels is 27% higher and in the mesentery is 400% higher of the older rats (24 months) compared with the younger rats (9 months) (76). In healthy elderly (≥ 75 years old), the mast cells in the skin increased by 40% compared with the biopsy of young people (≤30 years old) (77). Furthermore, although the liver has only a slight aging process compared to other organs, mast cells also play an important role in this process (32). One study has demonstrated that inhibiting SCF/c-Kit signaling pathway can reduce biliary senescence, with decrease mast cells activation and hepatic damage (78). In conclusion, the increase of mast cells can be detected in a variety of aging organs, so we suspect that they may play a role in the process of organ aging. The effect of mast cells on cardiac senescence can be reflected from two aspects: structure and function (79). It is normally assumed that the damage and apoptosis caused by mast cells to cardiomyocytes will eventually lead to cardiac dysfunction, a manifestation of cardiac aging. Co-culture of mast cells with cardiomyocytes promotes significant cardiomyocytes apoptosis for possibly the exposure to mast cell granules (80). It has been suggested that chymase derived from CMCs may induce myosin degradation in cardiomyocytes (81). Furthermore, activation of CMCs is pro-inflammatory and not only induces apoptosis, but also leads to extracellular matrix degradation, which may lead to eventual myocardial dysfunction (**Figure 2**) (82). These data suggest that CMCs can induce heart aging, but a more detailed mechanism remains to be explored.

## The trigger of CMCs activation and degranulation

Degranulation is considered to be the main way of mast cells playing physiological and pathological roles with IgE as the main trigger. As well known, mast cells express a large number of FcϵRI receptors, once IgE receptor cross-linking and calcium influx lead to mast cell degranulation (83). Currently, there are at least three ways in which mast cells release intracellular mediators, namely kiss-and-run, piecemeal, and compound exocytosis (84). In IgE-mediated allergic reactions, almost all vesicles are released from mast cells within minutes to hours. However, IgE is not the only trigger that stimulates mast cell degranulation, and activation induced by different components also leads to release of different mediators. There are numerous stimulants such as IgG, neuropeptides, cytokines, chemokines, TLR ligands, complements and other inflammatory products, that can directly cause mast cells to degranulate and selectively release mediators to stimulate proliferation, differentiation and migration (85). Mast cells subsets functions are different, not only because of the mediators produced, but also because of different sensitivities to stimulus. In addition to endogenous stimulus, some exogenous molecules can also directly activate mast cells, manifested as drug side effects or aggravating individual allergic state (86). It is worth mentioning that the process of mast cell degranulation in fibrosis is different from that in allergic reactions, and the release of mast cell vesicles may be more frequent and accompany with more subtle symptoms. It can occur by a slow process called piecemeal degranulation, and the vesicles can travel through the lymphatic vessels across the interstitial space to distant lymph nodes. Additionally, less discussed mechanism is the direct penetration of mast cell vesicles into another cell *via* intercellular contact, known as the transgranulation (83).

## CMCs in cardiac inflammation and functional remodeling following injury

The growing evidence shows that CMCs plays an important role in the occurrence and development of cardiovascular diseases (87). After myocardial infarction, CMCs density increase, rapidly degranulate, release a large number of bioactive mediators and initiate a cascade of cytokines to promote early inflammatory healing (88). CMCs play an undeniable role in the cardiac inflammation initiation and resolution. Because optimal healing requires inhibition of chemokine and cytokine synthesis, this leads to regression of inflammation and collagen deposition (31). However, the influence of CMCs on fibrosis remains a focus. CMCs produce a variety of growth factors, angiogenic factors and extracellular matrix regulators. All the products can affect matrix remodeling, promote granulation and scar formation, and have an important role on cardiac remodeling.

### Inflammatory Development and Resolution

The association of inflammation with myocardial infarction has been perceived for more than a century and inflammation is properly considered part of the healing process. The involvement of mast cells in inflammation has traditionally been thought to be only one aspect of the allergic response, but this does not seem to be the case. Following cardiac injury, the internal and external factors mentioned above can induce CMCs degranulation, and their derived histamine and TNF activate microvascular endothelium, up-regulate P and E-selectin, respectively, as well as adhesion molecules such as ICAM-1, which affect vascular tension and permeability. Eventually it mediates the infiltration of inflammatory cells, such as neutrophils, basophils, monocytes/macrophages, lymphocytes, etc (85) (89).

Inflammation benefits to cardiac repair, but this effect does not last (88). The release of cytokines and inflammatory cells infiltration directly or indirectly induced by CMCs are significant events in the progression of myocardial infarction, which play a key role in phagocytosis and clearance of dead cells and debris. Nevertheless, this acute inflammatory response is transient and then disappears (31, 90). This may be related to some anti-inflammatory mediators secreted by CMCs, such as IL-10 and IL-13, which can limit the expansion of inflammatory response and protect non-infarcted cardiomyocytes. IL-10 restrains the inflammatory response by inhibiting the production of IL-1α, IL-1β, TNF-α, IL-6, and IL-8 through lipopolysaccharide-activated monocytes (91). This can be demonstrated by the obvious inflammatory response of IL-10 knockout mice after myocardial infarction, which is characterized by increased neutrophil infiltration and elevated blood TNF-α levels (92). The importance of IL-13 on CMCs needs to be further investigated as it is not only derived from CMCs, but also secreted by many other cells in the microenvironment. In addition, mast cells can also exert anti-inflammatory or immunosuppressive effects by releasing mediators that degrade proinflammatory molecules (52). Mast cell proteinase 4 has been shown to degrade mast cell-derived TNF in mice *in vitro*, and it also can reduce TNF levels *in vivo* and limit inflammation (93). Besides, IL-37 is an important regulatory cytokine that inhibits inflammation, and mast cells can modulate the anti-inflammatory activity of IL-37 by trypsin-like action, resulting in the more biologically active form of IL-37 (94). Notably, VEGF-C is a major lymphangiogenic factor produced by human CMCs (95, 96), which has a potential cardioprotective effect, as cardiac lymphatic activation contributes to inflammation resolution and plays a crucial role in fighting myocardial edema (60, 97). Furthermore, mast cells can also inhibit inflammation through activation of PAR-2 on cardiomyocytes (64). Timely suppression of the inflammatory mediators such as chemokines and cytokines in healing infarction is critical to the repair process and can inhibit the continuous recruitment of inflammatory cells (31). More detailed anti-inflammatory mechanisms of mast cells remain to be studied. If we can find out the specific mechanism of the occurrence and resolution of cardiac inflammation regulated by CMCs, and then identify clinically appropriate targets, it may bring great improvement to the treatment of cardiovascular disease.

### CMCs: Pro-Fibrosis and Anti-Fibrosis

Although the obvious inflammation-related properties of CMCs, its main function in cardiac remodeling is related to the regulation of fibrous tissue metabolism. Cardiac fibrosis is actually an accumulation of the extracellular matrix, such as collagen (89). However, current studies have found that CMCs are double-edged sword in inducing cardiac remodeling, which can not only stimulate collagen synthesis and lead to fibrosis, but also induce matrix metalloproteinase activation and collagen degradation, with ultimately ventricular dilation (87).

**Figure 3:**
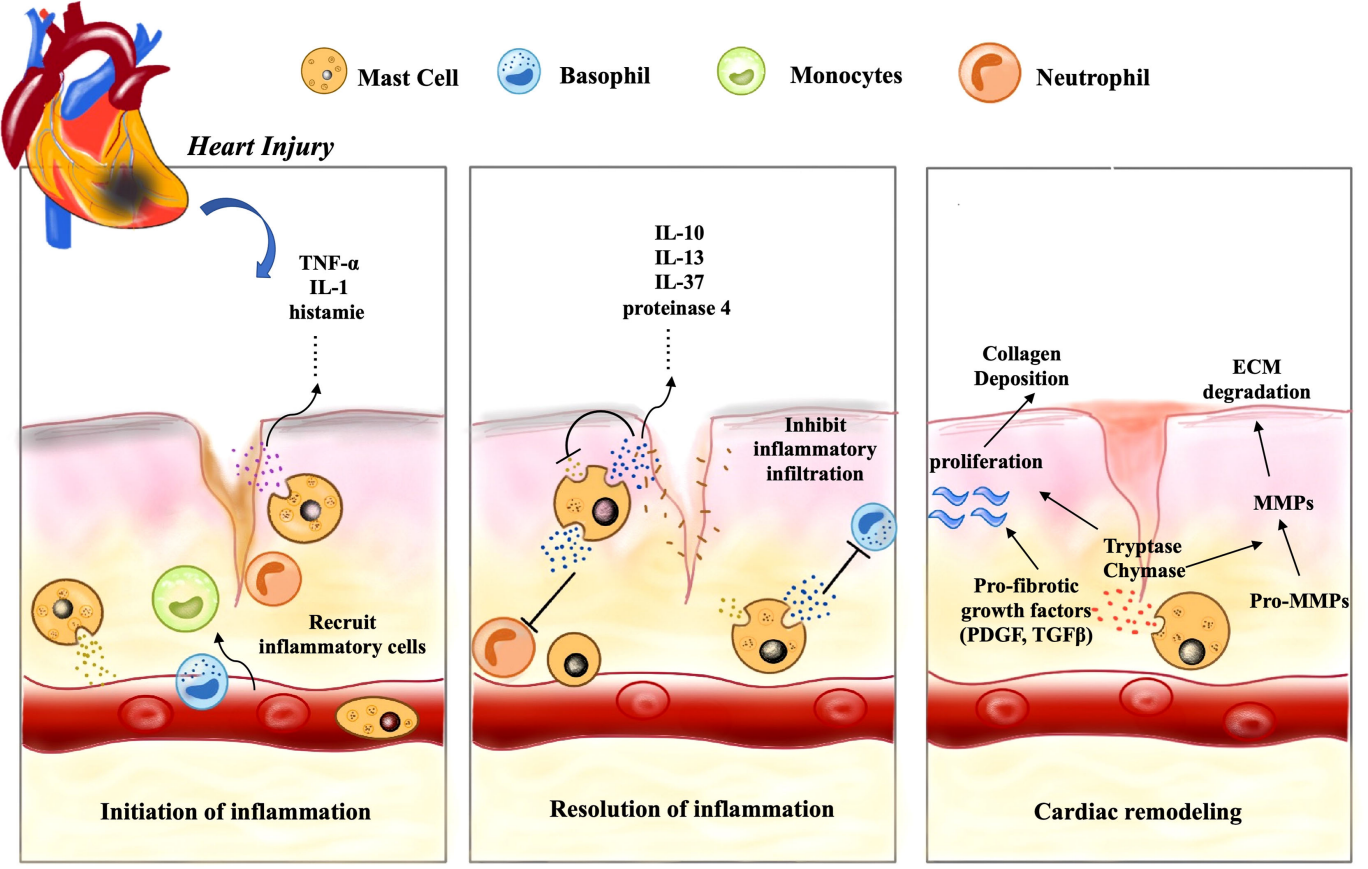
CMCs play an important role in cardiac remodeling following injury. Following cardiac injury, firstly, CMCs are activated, release a large number of inflammatory mediators, such as histamine, TNF-α and IL-1 to change vascular permeability, recruit a lot of inflammatory cells (basophils, monocytes, neutrophils, etc.), and induce early inflammation. Secondly, CMCs also mediate the inflammatory resolution by secreting some anti-inflammatory factors such as IL-10, IL-13 and IL-37. Finally, a series of pro-fibrotic and anti-fibrotic effects coexist and lead to cardiac remodeling.

Firstly, fibrosis is necessary for proper wound healing which can restores function to damaged tissue after myocardial injury, such as myocardial infarction or hypertension-induced stretch injury. Chymase and tryptase in CMCs have pro-fibrotic properties which are well-known fibroblast activity promoters, can mediate the activation of TGF-β and Ang II. However, fibrotic deposits are essential to restore normal heart function, but excessive remodeling can reduce contractile force and heart function, resulting in chronic heart failure (15). Additionally, CMCs can also secrete some anti-fibrotic mediators, such as IL-10, IL-13, CXCL-10 and VEGF, which have their own anti-fibrotic pathways, respectively (98). For example, IL-10 can reduce fibrotic remodeling by decreasing IL-1β and TNF levels, as well as MMP-9 expression and activity, and by increasing capillary density (99). CMCs-derived IL-13 can induce macrophages with an M2c phenotype, which is associated with reduced fibrosis. Moreover, VEGF-A can increase capillary density in damaged tissues and promote proper repair of cardiac fibrosis (93). At last, CXCL10 has been proved that it can inhibit the migration of fibroblasts to myocardium and delay their phenotypic differentiation into fibrogenic myofibroblasts. (**Figure 3**) (15). It cannot be ignored that CMCs have significant pro-fibrotic and anti-fibrotic effects, several studies have drawn controversial conclusions and described possible implications for this phenomenon, including harmful, neutral, or protective effects in cardiac remodeling (100). These conflicting conclusions are attributed to the failure to ensure a strictly correct clinical environment and the selection of appropriate animal models (27). Different culture systems, primary cell sources and even the initial cell number used in the experiment are also critical, and subtle differences may lead to different or even contradictory conclusions. To clarify these contradictory results, it is significant to correctly understand the characteristics of each *in vitro* and *in vivo* system used to culture mast cells, which can help us understand the real function of CMCs in the heart (21).

## Future perspective

Like macrophages and dendritic cells, CMCs are highly heterogeneous population of innate immune cells, with different morphological functions, mediator contents and surface receptors. The origin and differentiation of the different subsets remain unclear. CMCs are strategically located in close proximity to cardiomyocytes, coronary microvessels, nerves, and lymphatic vessels. Understanding the specific roles of CMCs in different sites of the heart in pathological and physiological processes will lead to a breakthrough in the treatment of cardiovascular diseases. Although they are distributed in small numbers and proportions within the steady-state heart, we reasonably suspect they are linked to the cardiac development and function, even the aging process. In a word, CMCs are a double-edged sword that may have potentially beneficial or harmful effects. The detailed roles of CMCs in cardiac development and injury remain controversial and contradictory, thus, several key questions about them remain unanswered. For example, the mechanisms about migration and differentiation of CMCs remain to be confirmed: whether CMCs precursors are regulated by specific mediators during migration to heart, whether CMCs proliferate and renew according to the pathway we mentioned above in both presence and absence of pathological injury, and whether cardiac-resident and recruited mast cells play divergent roles during homeostasis. Do different CMCs subsets have the same origin and developmental process, and whether their different phenotypes are changed by their microenvironment or driven by their designated progenitor cells? Specific mechanisms of CMCs on the development and function of the heart remain in the speculative stage. Its effect on the aging of the heart is only inferred from the performance of other organs. Some direct evidence is still lacking. With the growing understanding to CMCs, the other function may be demonstrated in future except for pro-inflammation and pro-fibrosis in cardiac injury. However, the dispute as to whether they perform harmful, neutral or protective activities has also not been resolved.

## Author contributions

JJ and YJ collected the material and draw Figures. JJ and ZS wrote the draft. ZS provides ideas and grant. SC revised the manuscript. All authors contributed to the article and approved the submitted version.

## Funding

This work was supported by National Natural Science Foundation of China (Grant No. 81871244), Primary Research & Development Plan of Jiangsu Province (BE2019700), Jiangsu Province “333” project (BRA2018016), Six talent peaks project in Jiangsu Province (2019-WSN-122), Projects of International Cooperation from Jiangsu (BX2019100), and international cooperation and exchange from Zhenjiang (GJ2020010).

## Conflict of Interest

The authors declare that the research was conducted in the absence of any commercial or financial relationships that could be construed as a potential conflict of interest.

## Publisher’s Note

All claims expressed in this article are solely those of the authors and do not necessarily represent those of their affiliated organizations, or those of the publisher, the editors and the reviewers. Any product that may be evaluated in this article, or claim that may be made by its manufacturer, is not guaranteed or endorsed by the publisher.
